# A Differentiated Discussion About AI Education K-12

**DOI:** 10.1007/s13218-021-00724-8

**Published:** 2021-05-13

**Authors:** Gerald Steinbauer, Martin Kandlhofer, Tara Chklovski, Fredrik Heintz, Sven Koenig

**Affiliations:** 1 Graz University of Technology, Graz, Austria; 2 Austrian Computer Society, San Francisco, Austria; 3 Technovation, San Antonio, USA; 4 Linköping University, Linköping, Sweden; 5 University of Southern California, Los Angeles, USA

**Keywords:** AI, K-12, Education

## Abstract

AI Education for K-12 and in particular AI literacy gained huge interest recently due to the significantly influence in daily life, society, and economy. In this paper we discuss this topic of early AI education along four dimensions: (1) formal versus informal education, (2) cooperation of researchers in AI and education, (3) the level of education, and (4) concepts and tools.

## Introduction and Motivation

Artificial Intelligence (AI) has gained significant influence across various sectors and fields and became a major topic of discussion. The impact of AI on the working world as well as on our everyday life poses a challenge for education systems. Sound knowledge about principles and concepts of AI, the ability to understand and use AI tools, techniques and methods, together with the ability to analyze and identify long-term benefits, social and ethical aspects of AI, are becoming key skills of the twenty-first century.

Teaching these AI skills has traditionally been done at the university level. In recent years several initiatives and projects have emerged which pursue the mission of AI education at the K-12 level.

In order to provide a differentiated view on the topic of educating younger people in AI we will look at the field from four angles. The first important angle is the relation between formal and informal education. This aspect is in particular important for the scalability of approaches. In particular, informal education gained a lot of attention during the COVID-19 pandemic. The second important aspect is the cooperation between skilled researchers from the field of AI and researchers from education as well as teachers in schools. This aspect affects the quality of the education as well as the potential outreach. The third angle we need to look at is the level of the AI education. Here we see a wide range from activities for a very broad audience to elite programs for gifted students close to academic courses with a strong focus on science. The fourth axis we need to look at are adequate concepts and tools for teaching AI to youngsters. While there is a rather clear picture of how AI should be taught at the university level, it is still under debate how AI can be taught at the K-12 level.

## Formal Versus Informal Education

In *formal education* we usually understand a learning process that is structured and systematic, is guided by educated teachers, takes place in class-rooms and institutions like schools or universities, and there are standards and certifications that are ruled by governmental bodies. In contrast, *informal education* is usually not bound to institutions or given structures and could happen at various places and in many forms.

Recently we observed big shift from formal to informal education. Due to recent advances in virtualization of teaching, numerous organizations like Udacity, Coursera or Udemy, to name a few, emerged providing informal education. Some of them are affiliated with top universities and provide high-end courses. This is a major transition that traditional educators like schools or universities need to recognize. A positive effect is that learning and technology will be in some way democratized.

When we look at AI education for K-12 we see both pillars. On one hand, there are numerous grass-root initiatives that offer material, courses, and training for AI in different quality, density, and quantity. Usually such activities move faster and are able to react to new topics, trends or needs much quicker. On the other hand, governments and supporting institutions and associations realized the necessity to integrate AI education in their national education systems and to provide more structure for AI at K-12 level in terms of curricula, guidelines, or standards. Obviously these activities are slower but gained more attention recently.

One example of formal education in AI for young people is the AI4K12 initiative [1]. The initiative is supported by the National Science Fund (NSF) and the Association for the Advancement of Artificial Intelligence (AAAI) and cooperates with the Computer Science Teachers Association (CSTA) which aims at improving the computer science education in schools by providing corresponding standards. AI4K12 aims at the development of national guidelines for AI education for K-12. Due to the institutional background and targeting teachers as valuable multipliers the activity has clearly a formal aim. An asset of this activity is the development of the standards around the *Five Big Ideas in AI* (perception, representation and reasoning, learning, natural interaction, and societal impact) [2]. These ideas cover a broad range of topics in AI very naturally. The developed guidelines nicely break down the ideas to topics that should be taught at different age groups and provide concepts and learning activities [3]. Moreover, it provides an online repository for teaching material. Visions of AI4K12 that are shared among many initiatives are (1) to educate citizens to understand AI and allow informed discussions and (2) to educate the AI-literate workforce of the future.

AI Singapore (AISG) is another formal approach to AI education. It was initiated by Singapore’s National Research Foundation and has the overall goal to improve the competitiveness of Singapore in AI. Besides, research and industry education plays a strong role. There are two programs related to AI K12 education. Both follow the train-the-trainer concept in order to improve the skills of teachers and to reach out into schools. The AI4Kids initiative aims at youngsters at the age between 9 and 12 years and focuses on basics in AI with a strong connection to coding [4]. Teachers can obtain certifications from introduction workshops. The initiative has a narrower scope compared to AI4K12, as the topics are more technology-oriented and no general curriculum is envisioned. The AI4Students initiative aims at students at high schools and colleges. This initiative cooperates with the company DataCamp and focuses mainly on big data and data analysis. The initiative acts as a kind of proxy for the existing courses of DataCamp and their learning analytical tools.

After the announcement of the *New Generation Artificial Intelligence Development Plan* by the Chinese government [5], a first textbook for AI education was published. The textbook comprises learning activities for a wide grade-band, from primary to secondary school. Details about the content of the textbook can be found in [6]. Yet, it is not sure if and how this activity is integrated into the official curriculum.

South Korea also follows the formal approach to support the competitiveness in AI by providing official guidelines and material. The learning is organized around key areas like understanding AI, AI and data or application of AI and is diversified for the individual grade-bands. In the guidelines there is a strong focus on data science and machine learning with a strong connection to coding as method of learning. A more detailed description of the South Korean initiative can be found in [6].

Australia follows a different approach to foster AI education for youngsters. Following the general Digital Technologies section of the Australian Curriculum, which defines in general the expectations on acquired skills of students of different grades, AI topics are mapped on that guidelines rather than developed as a standalone AI curriculum. Using the quite open description of national curricula with parts for understanding and building digital systems, concepts from AI like representations, data, or algorithms can easily be integrated. The MOOCS program of the Computer Science Education Research Group (CSER) provides a collection of introduction MOOCS to AI for teachers in primary and secondary schools.^1^ The courses are low-profile and aim to empower teachers to start teaching AI in school. As an introductory course the program has a strong focus on easily accessible topics from the machine learning area.

In the European Union (EU) education is a competence of the individual member states. In the area of AI there is a strong focus of the European Commission towards research and industry. In the *White Paper On Artificial Intelligence—A European approach to excellence and trust* education is mentioned in the sense of using AI to improve education as well as educate people to understand AI but no detailed plan for AI education K-12 is given. Although there is no development of curricula on the EU level there are initiatives like *AI Basis for Schools* that is integrated in the European CodeWeek that aim on training teachers in foundations of AI to allow them to integrate AI into their national curricula (to the extend the curricula allow such an integration).

A final example of an initiative to support formal education in AI in schools is the *European Driving License for Robots and Intelligent Systems (EDLRIS)* [7]. This European project followed the train-the-trainer approach to empower teachers to teach AI in classrooms. Within the project mini-curricula for Robotics and AI for a basic and advanced level had been developed. The intended duration of the courses based on the curricula are 5 days distributed over 3–5 weeks with alternating face-to-face and online units. Moreover, ready-to-use material for the different courses were developed comprising, for instance, background material, paper and pencil examples and coding examples. Furthermore, a certification system for teachers and students was developed. Certified teachers are entitled to give EDLRIS courses in their school as well as to use the provided material. Students who were educated in AI or Robotics by their teachers and had passed the certification can use this as an asset towards schools, universities or employers. This recognition, the openness of the Austrian computer science curriculum and the fact that EDLRIS courses are recognized as official training for the teachers, motivated numerous teachers to conduct a training.

An excellent example for informal education in AI K12 is the initiative Elements of AI (EoAI) [8]. The initiative provides a MOOC that covers foundations of AI and targets a broad general audience. It provides the basic module *Introduction of AI* and the more advanced module *Building AI*. While the first fosters a basic understanding and covers definitions of AI, basic methods and implications, the second aims at a deeper methodical and mathematical understanding of approaches and a connection to coding. Although the first module also covers problem solving more generally the initiative has a strong focus on machine learning and big data. The units usually contain accessible descriptions using mainly text plus some figures and formulae and are concluded with tests in form of multiple choice questions, interactive items or online coding examples. Users are also able to obtain a certificate. Initially the initiative was a national attempt of Finland. Meanwhile due to its simplicity, promotion on the European Level and translations to other languages, *EoAI* gained huge attention. Over several hundreds of thousands of users enrolled to the course and a good fraction obtained a certificate. The initiative is also inclusive as can be seen by a good mixture of gender, age and nationalities of the users.

A different informal approach is the Technovation AI Family Challenge initiative [9]. The central idea is to educate people in AI and to empower them to use this technology to solve societal challenges. The initiative aims at under-resourced communities and inclusion. Moreover, it fosters a strong participation of parents and guardians into the education process. More details about the initiatives can be found in [10]. The methodology of teaching follows the idea of solving a real word problem using AI. Thus, there is a startup phase were concepts of AI and coding are taught. Later, the participants select a challenge from their local community like fixing problems with the water supply and work on a solution. A motivation factor is that there is a competition for the most innovative solution. Due to support of donating companies and foundations the initiatives partner up with local ambassadors and coaches which drives the initiatives locally. Following this strategy, over 20,000 people registered and around half of them completed the challenge.

## Cooperation of AI Researcher, Education Science and Teachers

In general, researchers from the field of AI and learning sciences as well as teachers do not meet intensively. But the exchange and understanding among these groups is essential for the quality and therefore success as well as the sustainability of AI education for youngsters initiatives. While the topic of AI for education is a well-established field and researchers from learning sciences (education, psychology, neuroscience, linguistics, sociology, and anthropology) easily integrate results from AI research and communicate with AI researchers as well as teachers for decades [11], the topic of AI education for K-12 is a rather novel topic and fruitful alliances between AI researches on one hand and researchers from learning sciences and teachers on the other hand need to be established.

In times where most information and knowledge gets collected via informal challenges like social media, blogs, or online tutorials it is important to perform a quality check and feedback loop with persons that perform research in the core areas of AI, use AI methods to solve fundamental problems, and also look comprehensively into ethical and societal challenges of AI. AI is a big hype nowadays. Thus, there is a lot of half or wrong knowledge about the topic.

An early initiative for educating very young kids (K6) in AI was presented in [12]. Using the Scientists-in-Schools initiative of the Australian government. AI researchers teamed up with teachers in order to setup a challenging curriculum that present AI as a research discipline and its strong connections to fields like philosophy. In particular, teachers for the younger grades are still less good educated in science and technology and thus profit from such cooperation a lot.

The previously introduced Artificial Intelligence for K-12 initiative (AI4K12) [1] benefits from a strong cooperation with well-known researchers in AI and Cognitive/Social Robotics from institutions such as Carnegie Mellon University (CMU) or the Massachusetts Institute of Technology (MIT) as well as leading AI institutions such as the Association for the Advancement of Artificial Intelligence (AAAI). The AI4K12 Working Group is composed of practicing K-12 teachers that are supported by the AI experts. Following the Five Big Ideas the working group identified topics, concepts and skills that students in the different grade-bands should know [13].

Although it happens mainly within a single institution another good example for interdisciplinary cooperation is the project *Innovating Learning and Education in the Era of AI* at the MIT [14]. The project brings together researchers from various disciplines such as computer science, robotics, mechanical engineering, civil engineering, education, neuroscience and economics to create an open and innovative environment for the interaction of education and AI. The project is the umbrella for several smaller research projects to quickly but soundly assess ideas and ready-to-go AI learning units.

The European Driving License for Robots and Intelligent Systems project [15] also followed the way to team up people that conduct actual research in AI and Robotics with teacher training organizations for the development of the curricula. In contrast to AIK12 the AI and Robotics researchers played a more active role in the development by giving introduction lectures to the topics and by jointly developing examples with researchers from the education field. Regular testing workshops with teachers and feedback questionnaires ensured the pedagogical quality and maturity of the developed material.

Although AI education—in particular for the younger age group—are generally not in the focus of the top and most influencing conferences such as the International Joint Conference on AI (IJCAI), the AAAI Conference on AI, or the International Conference on Machine Learning (ICML) there are venues that aim at connecting cutting edge AI research with the education demands for AI. The Symposium on Educational Advances in Artificial Intelligence (EAAI) is an annual symposium collocated with the AAAI Conference on AI. It was held now for over a decade and mainly aims at issues that are related with AI education for undergraduates at university level and providing teaching material for that age group. Recently, the symposium opened its scope also into the direction of K-12 education.

Workshops devoted to AI education K-12 collocated with leading conferences in AI and AI in education such as the International Conference on Artificial Intelligence in Education (AIED) and IJCAI took place and showed the growing interest in this topic [16]. Also the well-established AAAI Spring and Fall Symposia series included tracks for AI K12 education lately. Also the Technical Symposium on Computer Science Education (SIGCSE) devoted a special session on AI K12 teaching [17].

## Ivory Tower Versus Everybody

When looking at the target audience and the particular goals of initiatives in AI K12 education there is a wide range from a very general audience to high potentials as well as from accessible introductions to elite programs. Many of these initiatives aim to foster a broad and open access which target in particular unrepresented persons or minorities but when it comes to activities with an advanced syllabus, resources like well-educated and motivated teachers or an adequate infrastructure play a crucial role.

Among other activities, AI4ALL provides the College Pathway program [18] that has a strong focus on AI as a career and on connecting high school students with universities in the area of AI. The program teams up with top US universities like Stanford, Carnegie Mellon or Texas A&M, provides a challenging AI education with close relation to actual AI research and allows for internships at the partner universities. Such a program is more likely accessible with an adequate academic and solid background. On one hand, it is a huge benefit for students if such institutions are involved and provide their expertise. On the other hand, it is also a good opportunity for these institutions to scout for young talents, which is a prominent concern of top universities these days.

Another example for advanced content are the MOOCs of the Hasso Plattner Institute [19]. The institute is organized as a foundation and aims at a founded and application-oriented education in computer science but also provides a strong outreach program organized as a kids academy. This activity in conjunction with the advanced MOOC program clearly aims at high potentials and gifted young people. On the other side of the spectrum we find Elements of AI that target a broad audience with its accessible curricula organized as a MOOC. The main aim is not to open an career path or to enable the founding of an AI start up but to provide a basic understanding of AI to as many people as possible.

The EDLRIS initiative addresses the the general public as well as the elite. The basic curriculum aims at providing basic knowledge of AI and enabling an informed reflection and discussion about the impact of AI. While the basic activities clearly aim at everybody, the advanced activities already aim to provide the basis for an extended education or career. Although the advanced curriculum demands some prerequisites by the participants it was still designed to be as open and accessible as possible. Unfortunately, practical experiences from the pilot phase showed that advanced education activities in AI for youngsters are only realizable if there is a proper academic background, adequate support by the schools and well educated and motivated teachers. Usually, such an environment can only be provided by privileged schools. Thus, democratization of education on the advanced level is much harder to achieve.

## Concepts and Tools

If one looks at methods and approaches focusing on teaching AI concepts at K-12 level, which comprises a wide age range from usually 6 to 18 years, the strong connection to the still ongoing personal and cognitive development of young people can be seen. While traditional AI education at undergraduate level is able to focus on teaching the algorithms and their background, in K-12 the building of models and a general understanding of things in a complex world matters a lot. Thus, in general many approaches follow the idea of Papert’s constructivism [20]. There are several good reasons for that. First of all, the basic idea of building an artefact supports the development of understanding. Moreover, when dealing with AI naturally the attribution of intelligent behavior to a machine appears. Thus, building or working with intelligent artifacts is a good way to structure teaching of AI concepts to youngsters [21]. The range of intelligent artifacts used is wide and may comprise smart speakers, educational robots, single-board computers, simulations, or interactive webpages. Furthermore, building such an artefact or at least interpreting its behavior fosters the understanding of its internal structure. This is of particular interest as many AI-enabled devices or services are black boxes hard to understand for non-experts.

Depending on the individual goal of an education activity, the scope of the activity, and the technical and content depth the educational means used may differ. The variety of means has a wide range: (1) unplugged activities [22], (2) videos, simulations or interactive presentations, (3) partly-finished projects or programming skeletons, and (4) full-fledged projects.

When looking at the creation of artefacts in the context of AI education immediately the question of a proper programming approach or language appears. A popular and intuitive option is a block-based programming environment that is easily accessible. Recently, for leading environments such as *Scratch* [23], the *MIT App Inventor* [24] or *Snap!* [25] extension blocks for AI had been developed [26]. Numerous tools developed for AI K12 education use this block-based approach and provide blocks that represent some AI methods. For instance, blocks for face or speech recognition using deep neural networks can be used as conditional blocks in programming. While blocks for machine learning approaches are very popular, blocks for symbolic AI can hardly be found. The block-based approaches try to hide the complexity of the underlying algorithms and code. For advanced curricula where more insight into the algorithms is indented, the programming language Python and the many related machine learning libraries and frameworks such as TensorFlow or PyTorch can be used. The flexibility is much higher so is the insight into the AI methods. Numerous examples and code skeletons can be found online. Finally, in the area of machine learning many tools and frameworks exist where no programming is needed at all. These tools aim at machine learning algorithms and focus on the training and evaluation of models. One example is Google’s Teachable Machine which is a web-based tool where models for machine learning can easily be configured and trained and the results can be visualized [27]. It clearly demonstrates the need for the collection and management of adequate training data. Another example is the TensorFlow Playground which provides an interactive and configurable model of neural networks [28]. Input data, network layout and network parameter can easily be adapted and the flow within the network and the results are directly visualized. The tool is very accessible but limited in the complexity of visualized networks and classification tasks compared to Python-based approaches in particular.

We close this section with some highlighted AI K12 education activities and tools. Heinze et al. presented an multi-year curricula for AI for the grade-band K1 to K6 [12]. A broad range of topics from history of AI to cognitive models are covered. The approach strongly follows the idea of constructivism and uses educational robots as artefacts. Fok and Ong reported in [29] from a quite advanced AI project in a high school. Following the project-based approach an industrial robot was turned into a Tic-Tac-Toe playing robot using a feed-forward neural network. The level of the complexity is impressive. Moreover, the realization of an intelligent opponent for game playing in the real world is a good basis to foster understanding of AI techniques.

The tool *AISpace2* by Zhou et al. [30] provides an online interactive representation of fundamental AI techniques such as Search, Planning, Constraint Satisfaction Problem (CSP), or Bayesian Networks. The system uses a web-frontend with JupyterLab for visualization and a Python-based backend using AIPython which implements a number of basic AI algorithms. The students can use different problem solving methods and a number of example problems are provided. Coppens et al. present in [31] a Virtual Reality (VR) framework for teaching the concept of reinforcement learning. The tool is based on navigation or treasure hunt in a labyrinth. The user is the learning agent. The environment plus the Q-values of particular actions (different direction in crossings) are visualized. The user neither sees the entire playing field nor the entire Q-table. By choosing and execution actions the Q-values are updated and give a hint for the next decision.

In [32] Featherston et al. use an Alice programming environment to set up a virtual educational robot that can be controlled by AI algorithms. The algorithm can be implemented using a graphical programming language. One asset of the framework is the coupling with the robot’s sensors and its behaviors. Johnson, et al. used a *System Engineering* approach to allow young students to understand the composition of complex systems [33]. The approach uses the flexible educational robot platform Lego Mindstorms and comprises analyzing as well as design challenges. Similar approaches also seem to be needed to understand AI systems although analyzing complex software systems is much harder than an electrical/mechanical system.

Reyes et al. [34] presented a curriculum for declarative programming. The objective of the curriculum is to allow students to solve problems using declarative programming. The 4 weeks course comprises lectures on the main basic concepts and assignments such as family hierarchy, map coloring or Sudoku. The course uses the modern Answer Set Programming (ASP) as a programming paradigm which has a strong connection to non-monotonic reasoning and constraint programming. Layer et al. reported in [35] on an intensive course aiming at topics from Computer Science including AI. One goal of the course design was to maintain the attention of the participants for a long time. Thus, the course design follows the concept “Inform, Experience, Implement”. First, students were introduced to the topic. This is succeeded by a phase where students work with a given artefact before the students are asked to create their own artefacts. Such a course layout might also be a blueprint for compact AI K12 education.

Following the idea that the interaction with a physical artefact is beneficial for the creation of a model of the artefact in particular for younger kids, Williams et al. developed the idea of PopBots [36]. The system is rather cost-effective and comprises a physical presence using Lego WeDo blocks and a tablet/mobile phone. The device runs the PopBlocks App that is a block-based programming language using only signs. Activities are grouped in the following categories: (1) *Knowledge-Based Systems* where examples for decision making are depicted, (2) *Supervised Learning* were examples for generating training examples are given and (3) *Generative AI* where the examples for generating items like songs are given. The interplay of the youngsters with the artefact and the example fosters the understanding of quite complex AI concepts. In [37] Touretzky introduced Calypso for Cozmo which is a rule-based language for the educational robot Cozmo that supports high-level concepts like speech recognition using cloud services, landmark-based navigation and behavior programming using state machines. Benefits of this programming environment in the context of AI education K-12 are that the execution of the rules can be visualized as well as explained and that the symbol grounding is done by the system allowing kids to address object or position in an abstract way (e.g. red cube), reducing the need to deal with coordinate frames.

## Conclusion

In this article we give an initial overview on the very active field of AI education for K-12. Although this topic did receive a lot less attention than AI education on the undergrad level, some research has been done in the last decades, but recently the research on the topic, the provided tools and the teaching initiatives exploded. This explosion is not only caused by the increased interest but also by the fact that informal approaches are more agile and allow the flourishing of a huge number of ideas and tools. Although, this drive is beneficial as it generates concepts that might fit a wide range of objectives, it makes the situation confusing, in particular for users end educators. Thus, repositories with material that is also assessed for some quality criteria as suggest by the AI4K12 are needed. One on hand, it allows educators to find good material more easily and on the other hand, it fosters the trust in the provided material. Such repositories are one pillar of the scalability of the idea of a broad and sound AI education for youngsters. Another pillar is the good mixture of formal and informal attempts. What is definitively missing is data of deployments of concepts with larger groups of participants and for a longer period of time (i.e. the entire grades). Many concepts and tools, also the ones presented in this overview article, are usually evaluated with a small group of kids (i.e. a class or club) and in a single shot. In order to judge the quality of proposed methods and tools and to extract best practice examples more founded evaluations are needed.

## Footnotes

1 https://csermoocs.adelaide.edu.au/available-moocs.
